# Social inequalities and the risk of multimorbidity in Mexican adults: an intersectional approach

**DOI:** 10.3389/fpubh.2026.1977366

**Published:** 2026-10-13

**Authors:** Dolores Mino-León, Hortensia Reyes-Morales, María Fernanda Carrillo-Vega, Héctor García-Hernández, Guillermo Salinas-Escudero

**Affiliations:** 1 Medical Research Unit on Clinical Epidemiology, Hospital de Especialidades Centro Médico Nacional Siglo XXI, Instituto Mexicano del Seguro Social, Mexico City, Mexico; 2 Center for Health Systems Research, Instituto Nacional de Salud Pública, Cuernavaca, Morelos, Mexico; 3 Research Division, Instituto Nacional de Geriatría, Mexico City, Mexico; 4 Center for Economic and Social Studies in Health, Hospital Infantil de México Federico Gómez, Mexico City, Mexico

**Keywords:** adult, health inequalites, MAIHDA, Mexico, multimorbidity

## Abstract

**Introduction:**

Multimorbidity, which is defined as the presence of two or more simultaneous diseases, is a growing global health problem that is influenced by social conditions, the interaction and overlap of which have been rarely recognized. Thus, this study aimed to analyze the impact of relevant social inequalities on the risk of Multimorbidity in Mexican adults with an intersectional approach.

**Methods:**

The data of 4,806 people older than 20 years who participated in the National Health and Nutrition Survey (ENSANUT) 2023 were analyzed. Multimorbidity was defined as the self-reported presence of two or more chronic diseases. A multilevel analysis of individual heterogeneity and discriminatory precision was used to identify intersectional inequalities.

**Results:**

The combinations of social attributes made up 38 strata. Social attributes explained 24.62% of the individual variability with respect to having Multimorbidity with an acceptable capacity to discriminate between having Multimorbidity or not. Young men who had higher education, employment and no social security had a lower probability of Multimorbidity; older women who had less education and no employment but did have social security were at greater risk. The main effects explained the total variance between strata, and important differences persisted between social combinations.

**Conclusion:**

This intersectional work reveals various social identities that are grouped into strata and represent patterns of social disadvantage associated with Multimorbidity.

## 1 Introduction

The study of the clinical condition of multimorbidity (MM), which is defined as the presence of two or more concurrent chronic diseases (CDs) (1), is of growing interest at the global level because it affects approximately one-third of the global population (2), and the prevalence of MM increases as populations age (3). The analysis of specific disease combinations has facilitated the identification of MM patterns (4, 5); from a clinical perspective, this provides valuable information for designing treatment and prognosis guidelines. However, these patterns alone do not explain the heterogeneity in the progression and health outcomes of patients. This has led to the study of MM with statistical models focused on people. Through this approach, it has been documented that the social inequalities of individuals with MM favor their mortality (6–8) and that inequalities are not universal for all types of MM (7). This information supports the assumption that the impact of inequality is heterogeneous within the categories of individuals with MM; that is, people have a particular level of health or disease that is determined by their social conditions. Since previous studies have only examined one or a few inequalities at the same time (9), some have evaluated MM by sex (10, 11), and the fact that they are the product of the interconnection and superposition of different social conditions has not been sufficiently addressed. The theory of intersectionality has facilitated a better understanding of the heterogeneity of the causal factors of health inequity from an approach that identifies the contribution of individual characteristics, represented differently between social groups according to the conditions of the context (9). A systematic review analyzed social determinants related with MM, identified 41 determinants, and were grouped into six domains: demographics, socioeconomic status, lifestyle, social networks and relationships, residential environment characteristics, and health service use (12). Using intersectionality as an analytical tool, the effects of these interconnections on health outcomes can be recognized (13). To contribute to the integrative analysis of the complexity of multicausality in health, this study aimed to investigate the impact of some social inequalities (age, sex, schooling, social security, and work) on the risk of MM in a population of Mexican adults through an intersectional approach.

## 2 Methods

### 2.1 Study population and database

Data from adults ≥20 years of age, obtained from the National Health and Nutrition Survey (ENSANUT) 2023 of Mexico, were analyzed. The objective of this survey, which has been carried out every 6 years since 2006 and annually since 2020 (ENSANUT Continua), is to determine the frequency, distribution, and trends of relevant indicators of health and nutritional conditions and their social determinants. The sampling is probabilistic and multistage (households, persons per household, and subsamples of children, adolescents, and adults). Health information and the use of health services are obtained through self-reports (14). In 2023, the survey included 6,672 people aged 20 and over and is available at https://ensanut.insp.mx/encuestas/ensanutcontinua2023/index.php.

### 2.2 Outcome variables

Information related to the reporting of suffering from any of the following CDs was obtained from the health questionnaire (diagnosis made previously by a physician): diabetes mellitus (DM), hypertension (HT), cardiovascular disease (CVD) (heart attack, angina, heart failure, stroke, or embolism), and kidney disease (KD) (recurrent urinary tract infections, kidney stones, kidney failure, or chronic kidney disease). With this information, individuals were classified into the following categories: having MM when a person reported suffering from ≥2 CDs or not having MM when they did not suffer from any of the abovementioned CDs.

### 2.3 Exposure variables

The following variables were selected as a proxy for the social determinants of health available in the 2023 ENSANUT continua survey: sex (men/women), age (20–59/60–79/≥ 80 years), schooling (up to complete high school/higher to complete high school), affiliated with a social security institution (yes/no), in Mexico, social security—in addition to the sickness and maternity component (which covers preventive medicine, medical care for illnesses, and disability benefits)—includes occupational risk insurance; disability and life insurance; insurance for retirement, advanced-age unemployment, and old age; as well as childcare services and social benefits (housing, education, and recreational and cultural facilities). Employment status (with/without paid work), indigenous language (yes/no), and type of locality [rural (< 2,500 inhabitants) /urban (2,500–99,999 inhabitants) / metropolitan (100 thousand and + inhabitants)].

### 2.4 Statistical analysis

Descriptive analysis of the variables was conducted; the means and standard deviations were used for continuous variables, and proportions were used for categorical variables. For the bivariate analysis, the *t* statistic was used for the continuous variables, and the chi-square test was used for the categorical variables; a value of *p* < 0.05 was considered statistically significant.

Then an intersectional analysis was performed, this methodology implies moving beyond class and socioeconomic position in analyzing the structural determinants of health, and on studying the strata defined by the combination of several socioeconomic dimensions (15), For the identification of intersectional inequalities, multilevel analysis of individual heterogeneity and discriminatory precision (MAIHDA) was used (16). Intersectional groups were defined according to the combinations of social attributes that were selected *a priori*, and two levels were established: individual and stratum (subgroups of individuals). Extensive details about the MAIHDA method have been described elsewhere (17–19).

### 2.5 Estimation of the number of intersectional strata

Based on the combinations of social attributes, sex (2 levels), age (3 levels), schooling (2 levels), social security (2 levels), and employment status (2 levels), 48 (2 × 3 × 2 × 2 × 2) potential strata were obtained. Of these, three were not formed because no individual had such combinations. Of the 45 remaining strata, seven were eliminated (10 people) for not having the minimum sample size (unweighted frequency of 5) to avoid compromising the precision of the model (20). The final analysis was carried out with 38 strata that included a total of 4,806 people.

The risk of MM was analyzed using two successive multilevel logistic regression models according to the procedures proposed by Evans and Merlo (16, 17).

#### 2.5.1 Model 1: null or unadjusted intersectional

This model provided information related to the general inequality of the sample and summarizes the degree of heterogeneity of the MM variable within and between strata through estimation of the variance partition coefficient (VPC). A high value indicates that there is similarity within the intersectional strata, but also a difference between them.

#### 2.5.2 Model 2: main effects

In this model, all social variables were included in an additive way (sex, age, education, social security, and work) as fixed effects. For each variable, the odds ratio (OR) and its 95% confidence interval (95% CI) were estimated. For the model, the VPC and the proportional change in variance (PCV) were calculated, which indicates the proportion of variance between the strata that is explained by the additive main effects; its value is expressed as a percentage, whereas 1-PCV represents the percentage of the variance at the stratum level that is not explained by additive effects and is due to multiplicative effects.

For the two models, the area under the curve (AUC) was calculated as a measure of discriminatory precision (21). All analyses were performed in Stata/SE^®^ 17.0 (Statacorp, College Station, TX, USA).

### 2.6 Ethics statement

The Research Ethics Committee (REC) of the National Institute of Public Health (INSP) is responsible for evaluating the ethical aspects of the National Health and Nutrition Survey (ENSANUT). This committee ensures that the research meets ethical standards, protecting the rights and wellbeing of participants. The REC reviews the questionnaires, interview procedures, and consent forms to ensure that valid informed consent is obtained, and that participant information is protected. More information can be found at https://www.insp.mx/planeacion/contacto-del-comite-de-etica.

## 3 Results

Data from 4,806 people ≥20 years were analyzed (Figure 1); the average age was 45 ± 16.6 years, the highest proportion of people were in the 20–59 years group, 57% were women, 62% had a high school level, just over half had social security, and 66% did not have a job. The bivariate analysis revealed statistically significant differences between the groups with and without MM in all the variables analyzed, with a higher frequency of women, a higher average age, a lower education, and a lower proportion of people without work, although there was greater social security coverage in the MM group (Table 1).

**Figure 1:**
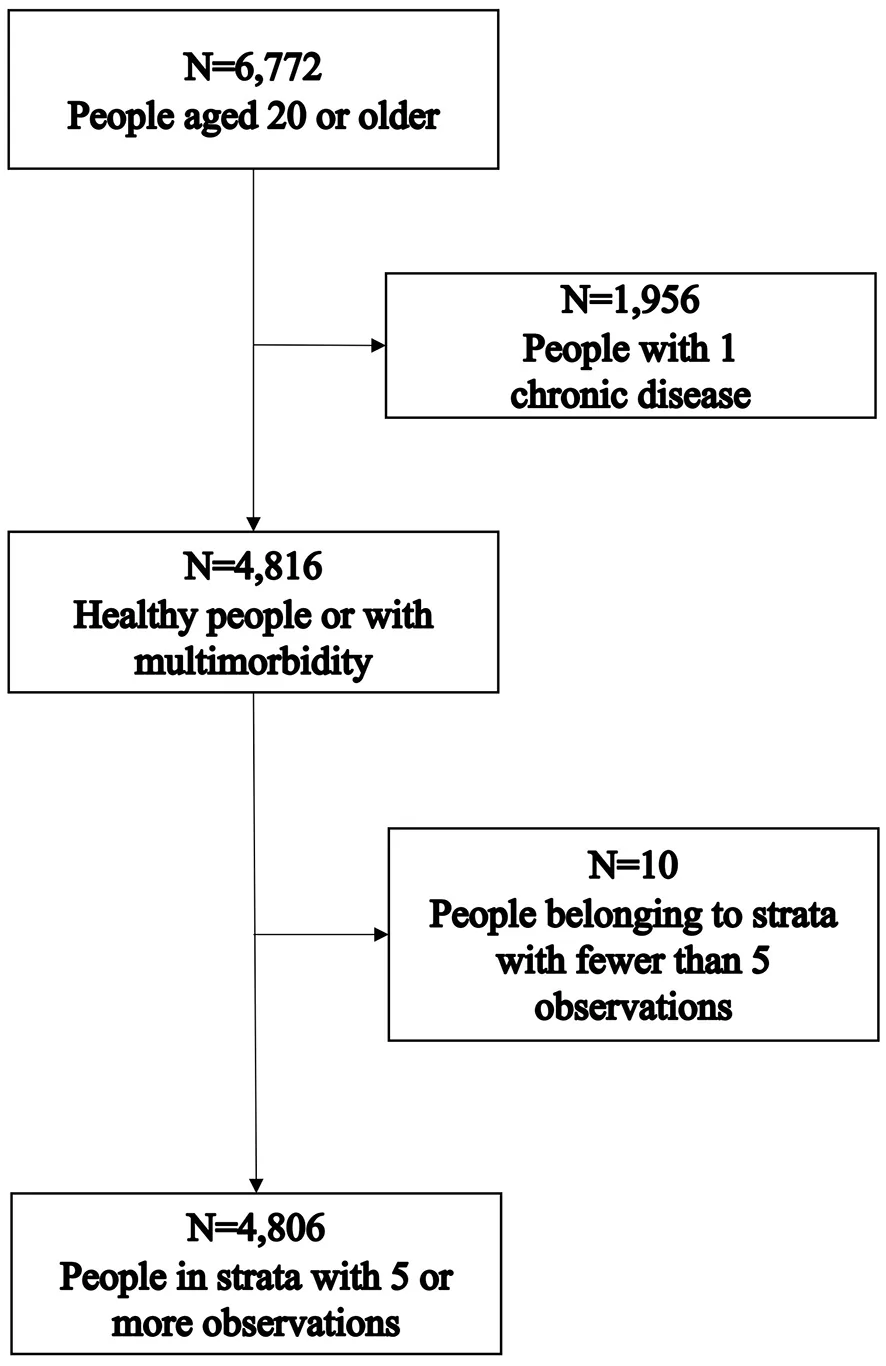
Flow chart of the ENSANUT sample continues 2023 and the sample analyzed.

**Table 1:** Characteristics of the study sample according to their multimorbidity condition.

Variable	Total		No multimorbidity		With multimorbidity		*p*
	*n* = 4,816		*n* = 3,820		*n* = 996		
	*n*	%	*n*	%	*n*	%	
Sex							
Men	2,098	43.6%	1,795	47.0%	303	30.4%	0.00
Women	2,718	56.4%	2,025	53.0%	693	69.6%	
Age (average ± SD^*^)	45.0	16.6	41.6	15.4	58.3	13.8	0.00
Age groups (Years)							
20–59	3,779	78.5%	3,273	85.7%	506	50.8%	0.00
60–79	916	10.7%	486	6.9%	430	28.6%	
≥80	121	2.5%	61	1.6%	60	6.0%	
Schooling							
Up to complete high school	2,969	61.6%	2,212	57.9%	757	76.0%	0.00
Higher to complete high school	1,847	38.4%	1,608	42.1%	239	24.0%	
Social security							
Without	2,253	47.3%	1,877	49.8%	376	37.9%	0.00
With	2,508	52.7%	1,892	50.2%	616	62.1%	
Work							
Without	1,659	34.4%	1,118	29.3%	541	54.3%	0.00
With	3,157	65.6%	2,702	70.7%	455	45.7%	
Indigenous language							
Yes	158	3.3%	130	3.4%	28	2.8%	0.35
No	4,658	96.7%	3,690	96.6%	968	97.2%	
Type of locality							
Rural (< 2,500 inhabitants)	1,217	25.3%	965	25.3%	252	25.3%	0.84
Urban (2,500–99,999 inhabitants)	1,646	34.2%	1,313	34.4%	333	33.4%	
Metropolitan (100 thousand and + inhabitants)	1,953	40.6%	1,542	40.4%	411	41.3%	

^*^SD, standard deviation.

### 3.1 Multilevel analysis of individual heterogeneity and discriminatory precision (MAIHDA)

#### 3.1.1 Model 1: null or unadjusted intersectional

The CPV value was 24.62% (95% CI: 16.2–35.5), which is classified as very good (22) and indicates that there is homogeneity in the presence of MM within the strata and heterogeneity between them. Furthermore, 24.62% of the individual variability with respect to the presence of MM can be explained at the level of the intersectional strata, that is, by the combinations of the social attributes that were analyzed. This is confirmed by the AUC value (75.64%), which is considered acceptable (21). to discriminate between people with and without MM (Table 2).

**Table 2:** Multilevel logistic regression models on the presence of multimorbidity.

Variable	Multimorbidity *n* (%)	Model 1 (intersectional not adjusted/null)		Model 2 (main effects)	
		OR	95% CI	OR	95% CI
Intercept		0.36	0.25–0.51	0.13	0.10–0.16
Sex					
Men	303 (14.4)			Ref.	
Women	993 (25.5)			1.97	1.66–2.34
Age groups					
20–59	506 (13.4)			Ref.	
60–79	430 (46.9)			4.63	3–88–5.53
≥ 80	60 (49.6)			4.45	2.95–6.71
Schooling					
Up to complete high school	757 (25.5)			Ref.	
Higher to complete high school	239 (12.9)			1.56	1.33–1.83
Social security					
Without	376 (16.7)			Ref.	
With	616 (24.6)			0.59	0.50–0.71
Work					
Without	541 (32.6)			Ref.	
With	455 (14.4)			0.68	0.57–0.80
VPC (95% CI)		24.62 % (16.2–35.5)		0.0 %	
PCV (95% CI)		—		100 %	
AUC (95% CI)		75.64 % (73.9–77.4)		75.03 % (27–76.8)	

#### 3.1.2 Model 2: main effects

In this model, the value of VPC fell to “0” with respect to the value of the null model, but the AUC remained (75.03%), which indicates that most of the differences in the individual start of the MM were not explained by additive main effects; instead, an acceptable discriminatory precision of the intersectional clustering was maintained. This means that the intersections can distinguish between the MM and not the MM. The 100% value of the PCV indicates that all the variance between strata is explained by the contribution of the main additive effects of the social variables and not by the interaction effect (Table 2).

Table 3 shows the strata with the lowest probability of having MM, mainly young men with higher education who are employed and not affiliated with social security. In contrast, the strata most likely to have MM were mainly composed of women, older individuals, those with lower education, those affiliated with social security, and those without work (Table 4).

**Table 3:** Intersectional strata that were less likely to have multimorbidity.

Strata ID	Men	Women	20–59 years	60–79 years old	≥80 years old	Up to complete high school	Higher to complete high school	Without SS^*^	With SS^*^	No work	With work	n	No. cases with MM^**^	OR	95% CI
1												276	13	4.9	4.0–6.0
2												37	3	7.1	5.5–9.0
3												417	31	7.4	6.2–8.9
4												451	36	8.0	6.7–9.4
5												213	20	9.2	7.6–11.0
6												43	5	10.6	8.5–13.2
7												43	5	11.3	9.2–13.9
8												323	38	11.9	10.1–14.0
9												131	17	13.0	10.8–15.6
10												375	51	13.7	11.7–15.9

^*^SS, Social security; ^**^ MM, multimorbidity. The shaded cells reflect the presence of the category in that strata.

**Table 4:** Intersectional strata that were more likely to have multimorbidity.

Strata ID	Men	Women	20–59 years	60–79 years old	≥80 years old	Up to complete high school	Higher to complete high school	Without SS^*^	With SS^*^	No work	With work	n	No. cases with MM^**^	OR	95% CI
29												20	8	42.3	36.5–48.3
30												48	21	44.1	38.7–49.6
31												24	11	47.1	37.4–57.0
32												96	46	48.1	43.1–53.1
33												57	30	52.0	46.6–57.4
34												25	13	52.9	42.9–62.6
35												124	67	53.8	49.2–58.4
36												61	34	55.2	50.0–60.3
37												38	24	63.7	54.2–72.2
38												182	118	64.6	60.6–68.4

^*^SS, Social security; ^**^ MM, multimorbidity. The shaded cells reflect the presence of the category in that strata.

## 4 Discussion

This analysis is one of the first efforts to examine the interconnections between different social variables on MM using a complex statistical design such as MAIHDA. The results provide a more detailed understanding of health inequalities through the identification of patterns of social disadvantage associated with MM in a sample of Mexican adults. Through this approach, social contexts are analyzed more than individual characteristics, which is part of the movement of “precision public health” (23), that acknowledges the limitations of employing a uniform strategy without taking into account the heterogeneity within and among populations.

The intersectional strata that were built and that were shown to have good discriminatory capacity facilitated the identification of conditions of vulnerability in the population from 60 years of age. The five strata with the highest risk of MM are women, people over 60 years of age, and those with education up to high school; in contrast, the lower risk strata are mostly men, young people and, those with education. A similar result was reported in a recent study in which the analysis of the intersectional strata revealed a lower probability of MM in men than in women, independent of the other social variables analyzed (24). However, since the interaction of sex and age with MM has rarely been studied (25), we believe that our study qualifies the risks between men and women that have been previously reported (6).

Another finding that was identified primarily in the strata of young adult men was their lower risk of MM, with a pattern of variable social disadvantages; however, among those with less education despite having social security and work, the risk of MM was greater. These data highlight the relevant role of education and its favorable impact on health, a result that is consistent with what was reported in a study of MM in the Mexican population and that used latent class analysis, in which the individuals of the class “healthy” were characterized by having a higher educational level (26). In high-income countries, a lower risk of MM has been demonstrated with higher education, which could be interpreted as an advantage for living conditions that are more favorable (27). However, in countries with less favorable social conditions, the results have not been conclusive (28). Thus, the findings of this study provide evidence that strengthens this association, even when different groupings with other characteristics are considered.

The main strength of the study is its intersectionality approach, which we used to quantitatively investigate disparities in presenting MM across strata with different combinations of socioeconomic dimensions. Consequently, we provide additional information on how this condition is distributed across groups defined by the intersection of multiple social characteristics, rather than focusing primarily on the independent associations of each characteristic with MM, as in other methods such as linear and logistic regression (29). This approach also shifts the focus from comparing MM disparities between the least and most disadvantaged groups to recognizing heterogeneity across specific intersectional profiles. Therefore, we provided a more complete understanding of which combinations of characteristics are associated with greater MM. The results can support the design of strategies to reduce the social disadvantages identified in the strata, thereby improving their effectiveness. In our research, for example, women aged 60–79 years, with incomplete high school and not working, constitute a profile with a higher prevalence of MM that must be considered a priority for intervention.

However, several limitations of this work should be considered. One is the use of secondary data which affects the possible number of strata. Furthermore, secondary analyses are not specifically designed for the objective of the work, so they are limited to the variables obtained in the original study. In this work, data on the social, economic (pension or retirement income), and health dimensions (CDs) were limited, and the primary information was obtained through self-reports. Also, it would be desirable to include more social variables in the analysis that may affect MM distribution. Nevertheless, including more variables requires a larger dataset to maintain adequate strata sizes.

## 5 Conclusion

This study revealed the distribution of MM risk among different strata in Mexico, a country with a high proportion of the marginalized and vulnerable population. The intersectional approach proposed in this study allowed identification of groups shaped by multiple social identities that intersect to determine the presence of MM.

## Data Availability

Publicly available datasets were analyzed in this study. This data can be found at: https://ensanut.insp.mx/encuestas/ensanutcontinua2023/descargas.php.
